# Reversible contrast enhancement for visualization of human temporal bones using micro computed tomography

**DOI:** 10.3389/fsurg.2022.952348

**Published:** 2022-10-04

**Authors:** Krishna K. Bommakanti, Janani S. Iyer, Varun Sagi, Alyssa Brown, Xiaojie Ma, Marissa Gonzales, Konstantina M. Stankovic

**Affiliations:** ^1^ Department of Head / Neck Surgery, University of California Los Angeles, Los Angeles, CA, United States; ^2^ Department of Otolaryngology – Head and Neck Surgery, Massachusetts Eye and Ear, Boston, MA, United States; ^3^ Department of Otolaryngology, Harvard Medical School, Boston, MA, United States; ^4^ Program in Speech and Hearing Bioscience and Technology, Harvard University Graduate School of Arts and Sciences, Cambridge, MA, United States; ^5^ Department of Otolaryngology – Head and Neck Surgery, Stanford University School of Medicine, Stanford, CA, United States; ^6^ University of Minnesota Medical School, Minneapolis, MN, United States

**Keywords:** micro-computed tomography, iodine staining, reversible, temporal bone, hearing loss

## Abstract

Sensorineural hearing loss (SNHL), which typically arises from the inner ear, is the most common sensory deficit worldwide. The traditional method for studying pathophysiology underlying human SNHL involves histological processing of the inner ear from temporal bones collected during autopsy. Histopathological analysis is destructive and limits future use of a given specimen. Non-destructive strategies for the study of the inner ear are urgently needed to fully leverage the utility of each specimen because access to human temporal bones is increasingly difficult and these precious specimens are required to uncover disease mechanisms and to enable development of new devices. We highlight the potential of reversible iodine staining for micro-computed tomography imaging of the human inner ear. This approach provides reversible, high-resolution visualization of intracochlear structures and is becoming more rapid and accessible.

## Introduction

Conventional temporal bone imaging approaches, including computed tomography (CT) and magnetic resonance imaging, reveal gross anatomic defects but provide limited resolution of intracochlear microanatomy due to the inner ear's encasement in the dense otic capsule. The gold standard for study of auditory pathology has long been *post-mortem* histology of temporal bones (1). This approach is time-intensive and requires several processing steps including fixation, decalcification, embedding, sectioning, and staining. These steps can introduce artifacts and limit the utility of sectioned temporal bones for other applications which require their three-dimensional structure. Since human temporal bones are precious specimens collected from autopsy, it is important to maximize their multi-utility (2). Therefore, a non-destructive imaging approach which provides structural information of the inner ear is invaluable in the study of human SNHL while allowing the imaged specimen to be used for additional downstream applications such as device development.

Recently, synchrotron-radiation phase-contrast imaging (SR-PCI) has been established as a high-resolution technique that can be used in non-decalcified, unsectioned, and unstained human temporal bones (3). While this method offers several advantages over traditional histology, access to SR-PCI infrastructure is limited and often tightly regulated (4). MicroCT, which affords low micrometer level spatial resolution, is a more widely available technology. MicroCT images have been used to develop high-resolution cochlear atlases from which lower resolution clinical CT images have been segmented into different cochlear regions (5, 6). Furthermore, microCT resolution can be improved with the use of contrast agents, of which iodine solutions, osmium tetroxide (OsO_4_), and phosphotungstic acid (PTA) are most commonly used (7, 8). Iodine staining is advantageous as it is not permanent and can be removed *via* leaching or chemical destaining with sodium thiosulfate (STS) (8). Combining the ease of access of microCT with improved resolution from iodine staining could provide a reversible and non-destructive imaging method for the study of temporal bones. We compare microCT images obtained with three different contrast agents and demonstrate the feasibility of reversible iodine staining for the first time in human temporal bones.

## Material and methods

### Specimen acquisition and preparation

Human temporal bones were procured at autopsy from donors aged between 6 and 90 years with unknown inner ear pathology. Collected bones bone were drilled to expose the round and oval windows, and excess bone was removed to meet the size limit of the microCT scanner. Bones were fixed in 10% neutral buffered formalin and later stored in Temple University Wetting Solution at 4°C prior to staining. The Massachusetts Eye and Ear permitted use of these deidentified specimens for research purposes.

### Staining with contrast agents

Three contrast agents - Lugol's iodine solution (I_2_KI), PTA, and OsO_4_ – were compared for their ability to stain soft tissue structures of the inner ear (7–9). An outline of the properties of each agent is provided in Table 1. Staining solutions were prepared by dissolving each compound in an aqueous solution at room temperature until saturation was reached. I_2_KI stain was prepared over multiple days to ensure proper solubility of iodine and potassium crystals. Once prepared, all stains were applied in the form of concentrated aqueous solutions in which the temporal bone samples were immersed. Additionally, round window membranes were penetrated with a hand-held 22-gauge needle and 2–3 ml of contrast agent was injected at a rate of 0.5 ml/min. The stapes footplate was removed to allow the oval window to serve as the egress point for the contrast agent.

**Table 1:** Overview of properties of staining and destaining agents.

Compound	Mechanism of action	Advantages	Disadvantages
Lugol's Iodine Solution (I_2_KI)	Iodine trimers bind to glycogen and lipids	• Provides best soft tissue contrast in non-decalcified specimens • Short incubation time • Non-toxic and water soluble • Reversible	• Risk of overstaining • No known histologic or fluorescent properties
Phosphotungstic acid (PTA)	Binds to fibrin, collagen, and fibers of connective tissues. Has electron-shell energies which match common x-ray source emissions	• Compatible with hematoxylin / eosin staining • Non-toxic	• Long incubation period • May not fully penetrate deep tissue layers
Osmium Tetroxide (OsO_4_)	Reacts with unsaturated fatty acids and increases x-ray absorption of cellular membranes	• Best source of contrast for nerve fibers • Commonly used post-fixative agent for staining	• Toxic • Not reversible • Requires decalcification for full effect
Sodium Thiosulfate (STS)	Reduction of halogenic compounds	• Removes iodine • Does not alter cellular morphology • Non-hazardous • Affordable	• May not fully penetrate deep tissue layers

Each specimen was stained for 48 h followed by a 24 h washing period in either distilled water (I_2_KI, OsO_4_, STS) or 70% ethanol (PTA). The specimens were placed on a shaker at 4°C for the duration of this time. Specimens were then imaged in respective washing solutions and returned to staining solution for additional 48 h intervals up through 240 h, again on a shaker at 4°C. Optimal staining duration for each agent was determined by visual observation of microCT images as well as calculated contrast ratios. Detailed stain preparation information is provided in Supplementary Data Sheet S1.

### Reversible I_2_KI staining

Previous studies have demonstrated that iodine-based contrast agents can be removed through leaching or chemical destaining (8). Leaching is a process by which iodine is removed *via* an osmotic gradient between stained specimen and surrounding fluid. This process is slow and requires replacement of leaching solution as it becomes saturated with iodine. Efficacy of leaching is variable and requires many weeks for full effect. An effective alternative is chemical destaining with STS (8).

In solution, STS reacts with dissolved iodine and reduces it to iodide which is transparent (8). The concentration of STS should be maintained at a high enough level to bind and convert iodine ions. However, concentrations ≥10% weight/volume of STS can alter properties of fixed specimens rendering them softer and less contrasted than the original specimen (8). Therefore, a 3% aqueous solution of STS was prepared to maintain a balance between effective iodine removal while limiting alteration of bony or soft tissue elements. Specimens were immersed in 15 ml of STS solution following completion of I_2_KI staining. The specimens were placed on a shaker at 4°C for the duration of this time. STS solution was replaced at 24 h intervals. Detailed solution preparation information is provided in Supplementary Data Sheet S1.

### Image acquisition and post-processing

Specimens were scanned using a Nikon Metrology HMX ST 225 scanner at the Center for Nanoscale Systems (Harvard University) in Boston, MA. This system uses a 225 kV microfocus x-ray source with 3 µm focal spot size. Pixel sizes range from 5 to 300 μm and the practical limit of resolution for images from this scanner is approximately 11 μm. All stained specimens were imaged at 90–92 kV and 92–108 mA for 14–21 μm voxel sizes. STS treated bones were imaged at 65 kV and 58 mA for a 19 μm voxel size. Ring-artifact-reduction was applied to all specimens. Due to intrinsic differences between the specimens and the staining properties, the imaging parameters were modified to optimize the resolution of each image. To ensure that the image quality was standardized, a histogram range of 8,000–16,000 pixels for image brightness was maintained. Images made on the Nikon Metrology HMX ST 225 were reconstructed using VGStudio viewing software and were stored as TIFF image stacks. Imaging parameters are described in Supplementary Table S1.

All images were reconstructed using Amira 6.4 (Thermo Fisher Scientific – FEI Visualization Sciences Group, Mérignac Cédex, France). Virtual sections were inspected for image contrast and visibility of selected soft tissue structures of the inner ear. In addition, x-ray densities for different regions including auditory nerve (AN), fluid-filled space, and bone were measured to quantify the staining for different contrast agents.

## Results

**Figure 1:**
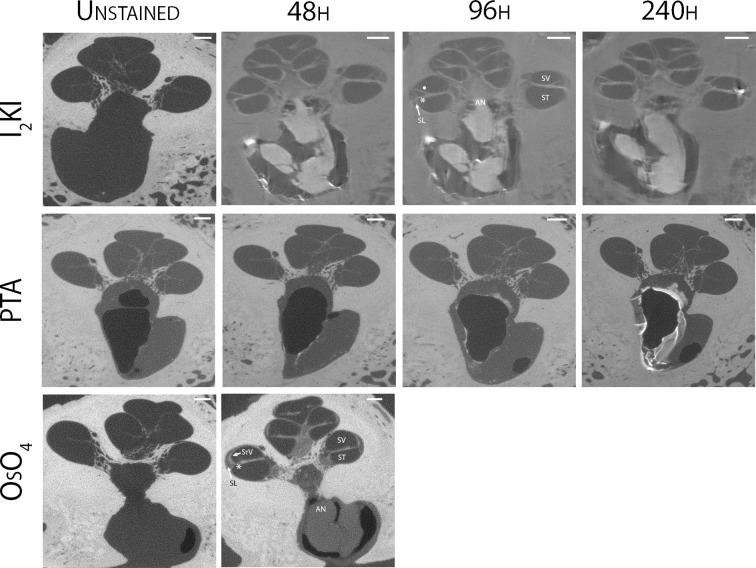
Comparison of uCT images obtained with different contrast agents at different timepoints. Mid-modiolar 2D virtual cross sections through the non-decalcified, non-dehydrated human cochlea. I_2_KI and PTA staining was continued through 240 h. OsO_4_ staining was completed only at the 48 h timepoint. Resolution of intracochlear structures including basilar membrane (*), Reissner's membrane (•), spiral ligament (SL), stria vascularis (StV) and the auditory nerve (AN) is highest in I_2_KI and OsO_4_ stained bones. SV, scala vestibuli; ST, scala tympani; I_2_KI, Lugol's iodine solution; PTA, phosphotungstic acid; OsO_4_, osmium tetroxide. All scales = 1 mm.

Mid-modiolar virtual two-dimensional (2D) sections through non-dehydrated, non-decalcified specimens were compared across each of the contrast agents at different time points (Figure 1). Additionally, contrast ratios were calculated for the AN relative to bone and fluid-filled spaces (Table 2). I_2_KI staining effectiveness peaked between 48 and 96 h after which soft tissue became saturated as evidenced by bright spots of iodine buildup. In I_2_KI stained bone, the basilar membrane (BM), Reissner's membrane (RM), and spiral ligament (SL) were visualized in the basal, middle, and apical turns of the cochlea. Clear distinctions between the scala tympani (ST) and scala vestibuli (SV) were also appreciated. Furthermore, the AN and its branching structure is seen. PTA staining, conversely, showed poorer resolution of intracochlear structures and the AN than I_2_KI staining at all time points. Faint contrast is visible at 96 h, at which point the BM can be partially visualized. Contrast enhancement did not improve appreciably at later time points. OsO_4_ stained bone was imaged at a single 48 h timepoint following the protocol adapted from Glueckert et al., 2018. In OsO_4_ stained bone, the BM is visualized in all cochlear turns, but the RM is only weakly observed in the basal turn. The stria vascularis (StV) and SL are well defined in the basal and middle turns, and the AN trunk is clearly visualized.

**Table 2:** Comparison of gray scale values for auditory nerve, fluid filled spaces, and bone at optimal time points for each solution.

Specimen	Solution	Timepoint (hrs)	AN Mean	Fluid Mean	Bone Mean	AN:Fluid	AN:Bone
1	None	0	N/A	11,306.771	24,048	-	-
	I_**2**_KI	96	10,447.72	9,227.59	9,672.5	1.132	1.08
	STS	48	5,819.98	5,680.01	10,885	1.025	0.535
2	None	0	N/A	11,295.64	23,632.5	-	-
	OsO_**4**_	48	16,171.85	13,167.78	27,574.5	1.228	0.586
3	None	0	N/A	8,460.89	17,733	-	-
	PTA	240	7,368.99	7,602.25	13,546.5	0.969	0.544

AN, auditory nerve; I_2_KI, Lugol's iodine solution; STS, sodium thiosulfate; OsO_4_, osmium tetroxide; PTA, phosphotungstic acid.

**Figure 2:**
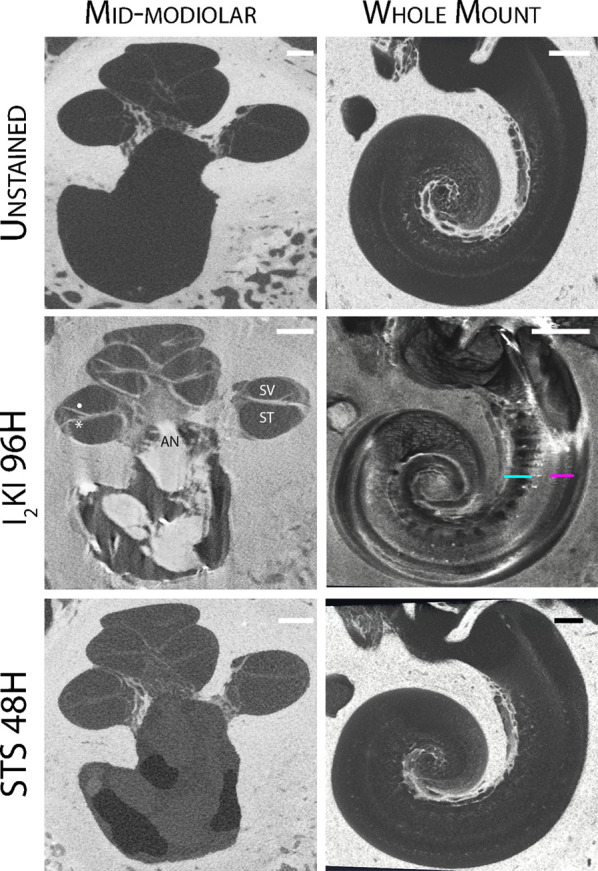
Reversible iodine staining of temporal bones. Mid-modiolar 2D virtual cross sections are displayed next to virtual cochlear wholemounts. Staining with I_2_KI greatly enhances the resolution of intracochlear structures. In the mid-modiolar view of I_2_KI stained bone, basilar membrane (*) and Reissner's membrane (•) are visualized with clear distinctions between scala tympani (ST) and scala vestibuli (SV). In the virtual wholemount, distinct cytoarchitecture of the modiolar core is appreciated, including auditory nerve (AN) fiber bundles (teal underline), and the organ of Corti containing hair cells (pink underline). These features are no longer visualized following destaining with STS for 48 h. I_2_KI, Lugol's iodine solution; STS, sodium thiosulfate. All scales = 1 mm.

Chemical destaining with STS was effective in removing I_2_KI from temporal bone specimens (Figure 2). The structural details of the cochlear interior and AN were no longer appreciated in the mid-modiolar virtual 2D sections after 48 h of treatment with STS.

Virtual whole mount of I_2_KI stained bone displayed the full cytoarchitecture of the organ of the Corti including the modiolar core, hair cells, and AN bundles. None of these features were visualized following destaining with STS.

**Figure 3:**
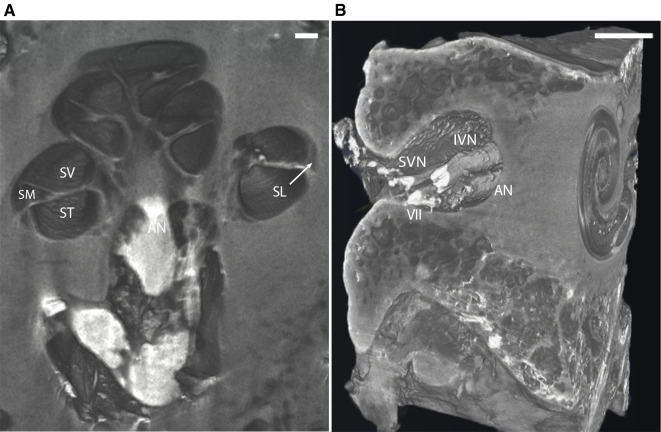
(**A**) Mid-modiolar 3D virtual cross-section through the human cochlea in I_2_KI stained bone. Boundaries between scala tympani (ST), scala vestibuli (SV), and scala media (SM) are appreciated. Spiral ligament (SL) is well defined, and auditory nerve trunk is seen. Scale = 1 mm. (**B**) 3D reconstruction of I_2_KI stained temporal bone. Anatomical relationship between the cochlea and the structures of the internal auditory meatus including facial nerve (VII), auditory nerve (AN), superior vestibular nerve (SVN) and inferior vestibular nerve (IVN) are seen. I_2_KI, Lugol's iodine solution. Scale = 0.5 cm.

Virtual 3D mid-modiolar section of I_2_KI stained bone provided distinct boundaries between the ST, SV, and scala media (SM) (Figure 3A). The SL and the AN are also well defined. 3D reconstruction of the I_2_KI stained temporal bone shows the anatomical relationship between the superior and inferior vestibular nerve, facial nerve, and AN in the internal auditory meatus (Figure 3B).

## Discussion

Human temporal bones are invaluable for the study of diseases of the auditory and vestibular systems, for research involving new devices and for training of surgeons and investigators alike. Nevertheless, resources devoted to the study of temporal bones have decreased over the years. In fact, only three federally-sponsored active temporal bone research laboratories remain today in the United States due to the high costs associated with collecting and processing these specimens (10). Moreover, collection and study of temporal bones from patients with well documented otologic disease and/or prior surgical procedures is limited. Of the nearly 200 deafness causing genes, histopathologic analysis has only been reported for 22 (11). This lack of reported histopathology extends to those who have undergone surgical procedures such as labyrinthectomies or cochlear implantation (10).

The scarcity of temporal bones characterized at the otopathological level can partially be attributed to the challenges of traditional histological processing. This approach is technically rigorous, and processing steps take many months. Additionally, histological processing can introduce artifacts into tissue which can confound findings (12). Furthermore, the 3D relationships between structures cannot easily be appreciated with sectioned temporal bone slides. These factors underly the need for alternative methods of studying auditory pathology that are faster, more accessible and cost-effective while facilitating multi-utility of the same specimen.

In this study, we demonstrate the feasibility of a non-destructive approach to studying human temporal bone histopathology using microCT technology coupled with iodine contrast. MicroCT is a readily available imaging modality that has been used extensively in the study of human temporal bones (13–15). Given comparable staining outcomes to OsO_4_, I_2_KI affords the additional benefit of reversibility. We have demonstrated that chemical destaining with STS returns I_2_KI stained bone to a near original state with regards to imaging properties. It is important to note, however, that destaining does not return a specimen to its initial *chemical* state as colorless iodine remains in the sample. Even so, the structural integrity of the specimen is left intact allowing for continued use of these bones for additional analyses.

The current study does have limitations inherent to the use of microCT imaging. Specifically, non-contrast microCT affords poor soft tissue visibility due to weak absorption of x-rays, as was evident in our unstained temporal bones. However, visualization of intracochlear structures was improved with contrast agents OsO_4_ and I_2_KI. A limitation of our methodology is that direct injection into the RW might cause injury to the cochlear cytoarchitecture, despite the injections being done in a controlled fashion by a skilled investigator. Finally, to maximize resolution provided by the microCT system, temporal bones need to be drilled down to the level of the otic capsule, which adds time prior to imaging.

## Conclusion

Temporal bones have been used in applications ranging from uncovering molecular and cellular mechanisms of audiovestibular dysfunction (11, 16–18), development of devices for inner ear stimulation and monitoring (19, 20), and validation of novel imaging strategies (3, 21–24). Therefore, the value of each bone cannot be understated. While we await the development of methods for investigation of temporal bones which preclude the need for processing, reversible I_2_KI staining with microCT can be used as an alternative approach for the study of the inner ear which preserves three-dimensional structure.

## Data Availability

The original contributions presented in the study are included in the article/**Supplementary Material**, further inquiries can be directed to the corresponding author/s.
