# Supplementary material for: Reversible contrast enhancement for visualization of human temporal bones using micro computed tomography
Source: Front Surg. 2022 Oct 4;9:952348. doi: 10.3389/fsurg.2022.952348 (PMC9577409; doi:10.3389/fsurg.2022.952348)
Supplement: Supplementary file 1 [file DataSheet1.docx]

**Supplemental Data Sheet S1**

**Stain preparation**

Preparation of OsO_4_ - 2% OsO_4_ (w/v)

1. Pulverize two 1 g ampules of osmium crystals
2. Dissolve in 200 mL of distilled water in disposable beaker
3. Place on stir plate for 24-48 hrs until stain appears homogenous and fully dissolved

Preparation of 200 mL stock solution of Lugol’s stain (I_2_KI) – 0.5% I_2_ (w/v), 1% KI (w/v)

1. Dissolve 2 g KI in 10 mL of distilled water
   1. Cover bottle in foil to prevent exposure to light
   2. Stir overnight
2. The next day pulverize 1 g I_2_ and add to solution above
   1. Allow this to stir for at least a few hours (until appears homogenous)
3. Add remaining 190 mL of distilled water and stir overnight
4. IMPORTANT: Keep bottle covered in foil
5. Once fully dissolve, filter solution into fresh bottle using 9 cm filter
6. Cover in foil and store at room temp

Preparation of 200 mL 0.3% PTA (w/v) in 70% EtOH

1. Mix 60 ml 1% PTA solution + 140 ml absolute ethanol to make 0.3% PTA in 70% ethanol

Preparation of 3% (w/v) sodium thiosulfate in dH_2_O

1. Dissolve 9 g of sodium thiosulfate in 300 mL distilled water
2. Stir at room temp until fully dissolved

**Human temporal bone staining**

Osmium staining of human temporal bones

1. Place temporal bone specimen in 70 mL of 2% OsO_4_ for 48hrs
2. Rinse sample in 70 mL of distilled water for 24 hrs until excess OsO_4_ is removed
3. Image sample in distilled water

PTA staining of human temporal bones

1. Place temporal bone specimen in 70 mL of 0.3% PTA in EtOH for 48 hrs
2. Rinse sample in 70 mL of 70% EtOH for 24 hrs
3. Image sample in 70% EtOH
4. Place sample in 70 mL fresh 0.3% PTA in EtOH for 48 hrs and repeat steps 2-3

Lugol’s stain (I_2_KI) staining of human temporal bones

1. Place temporal bone specimen in 70 mL of 0.5% I2, 1% KI in dH_2_O for 48 hrs
   1. ***Replenish with 70 mL of fresh 0.5% I_2_, 1% KI daily***
   2. ***Container should be wrapped in foil***
2. Rinse sample in 70 mL distilled water for 24 hrs
3. Image sample in distilled water
4. Place sample in 70mL fresh 0.5% I2, 1% KI in dH_2_O for 48 hrs and repeat steps 2-3
