# Supplementary material for: Reversible contrast enhancement for visualization of human temporal bones using micro computed tomography
Source: Front Surg. 2022 Oct 4;9:952348. doi: 10.3389/fsurg.2022.952348 (PMC9577409; doi:10.3389/fsurg.2022.952348)
Supplement: Supplementary file 2 [file Table1.docx]

Supplemental Table 1| Imaging parameters for temporal bones

| **Specimen** | **Stain** | **Timepoint (hrs)** | **Voltage (kVp)** | **Current (µA)** | **Final image resolution (µm)** |
| --- | --- | --- | --- | --- | --- |
| **1** | None | 0 | 97 | 92 | 18.71 |
|  | I_2_KI | 48 | 92 | 91 | 19.64 |
|  | STS | 48 | 58 | 65 | 19.41 |
|  | I_2_KI | 96 | 96 | 91 | 19.63 |
|  | I_2_KI | 240 | 92 | 90 | 20.12 |
| **2** | None | 0 | 95 | 90 | 14.6 |
|  | OsO_4_ | 48 | 103 | 92 | 15.1 |
| **3** | None | 0 | 100 | 90 | 14.8 |
|  | PTA | 48 | 108 | 92 | 16.04 |
|  | PTA | 96 | 108 | 90 | 17.14 |
|  | PTA | 240 | 97 | 91 | 17.07 |

kVp, kilovoltage peak; µA, microampere; µm, micrometer; I2KI, Lugol’s iodine solution; STS, sodium thiosulfate; OsO4, osmium tetroxide; PTA, phosphotungstic acid.
